# Social determinants of health correlations and resource usefulness at a Milwaukee free clinic for uninsured individuals: A cross-sectional study

**DOI:** 10.1017/cts.2024.503

**Published:** 2024-04-25

**Authors:** Jessica Miller, Adrianna Doucas, Hamsitha Karra, Suma K. Thareja, Owen Bowie, Xiaowei Dong, Jennifer Terrell, Samuel Hernandez, Ana Mia Corujo-Ramirez, Nicole Xia, Sabrina Qi, Chiang-Ching Huang, Rebecca Lundh, Staci A. Young

**Affiliations:** 1 Medical College of Wisconsin, Milwaukee, WI, USA; 2 Saturday Clinic for the Uninsured, Medical College of Wisconsin, Milwaukee, WI, USA; 3 University of Wisconsin-Milwaukee, Milwaukee, WI, USA

**Keywords:** Social determinants of health, primary care, uninsured, cross-sectional study, dental, insurance, free clinic, student-run clinic

## Abstract

**Introduction:**

Addressing social determinants of health (SDOH) is fundamental to improving health outcomes. At a student-run free clinic, we developed a screening process to understand the SDOH needs and resource utilization of Milwaukee’s uninsured population.

**Methods:**

In this cross-sectional study, we screened adult patients without health insurance (*N* = 238) for nine traditional SDOH needs as well as their access to dental and mental health care between October 2021 and October 2022. Patients were surveyed at intervals greater than or equal to 30 days. We assessed correlations between SDOH needs and trends in patient-reported resource usefulness.

**Results:**

Access to dental care (64.7%) and health insurance (51.3%) were the most frequently endorsed needs. We found significant correlations (*P* ≤ 0.05) between various SDOH needs. Notably, mental health access needs significantly correlated with dental (*r* = 0.41; 95% CI = 0.19, 0.63), medications (*r* = 0.51; 95% CI = 0.30, 0.72), utilities (*r* = 0.39; 95% CI = 0.17, 0.61), and food insecurity (*r* = 0.42; 95% CI = 0.19, 0.64). Food-housing (*r* = 0.55; 95% CI = 0.32, 0.78), housing-medications (*r* = 0.58; 95% CI = 0.35, 0.81), and medications-food (*r* = 0.53; 95% CI = 0.32, 0.74) were significantly correlated with each other. Longitudinal assessment of patient-reported usefulness informed changes in the resources offered.

**Conclusions:**

Understanding prominent SDOH needs can inform resource offerings and interventions, addressing root causes that burden under-resourced patients. In this study, patient-reported data about resource usefulness prompted the curation of new resources and volunteer roles. This proof-of-concept study shows how longitudinally tracking SDOH needs at low-resource clinics can inform psychosocial resources.

## Introduction

Screening for social determinants of health (SDOH), which are the conditions in which people live that impact their health outcomes and functioning, enables the delivery of comprehensive medical care and decreases morbidity and mortality [1]. Many studies also show that individuals from low-income backgrounds face disproportionate SDOH challenges, such as decreased access to stable housing, healthy food, and educational and employment opportunities [2–4]. Addressing SDOH needs is especially critical in clinics serving uninsured patients, as this population disproportionately faces barriers to healthcare [5]. In 2020, 9.7% of the United States population lacked health insurance [6]. Uninsured patients unequally suffer from chronic conditions [7,8], social barriers [7–14], and challenges in accessing healthcare [15–17], and yet, are historically underrepresented in studies [10,12,15].

Although the importance of screening for SDOH is established, many primary care clinics continue to face complex barriers regarding screening implementation and methodology [18–24], including a lack of standardization, time, personnel, and technological capability [15,25–27]. As an alternative to hiring and training screening personnel, electronic health records (EHRs) can be bootstrapped to integrate provider orders and web-based referrals [19,28]. However, many free or charitable clinics, such as the one where this study was conducted, cannot afford EHR platforms with this capability.

Some medical clinics assessed SDOH needs and resources offered among their respective patient populations [19,28], but they failed to report upon whether patients found the psychosocial resources they were given to be useful. Thus, an easy-to-implement, adaptable, and consistent process for assessing SDOH and resource usefulness in under-resourced primary care clinics is still lacking.

Since free clinics are designed to bridge the healthcare gap for uninsured individuals [29] and they primarily rely on private donations and volunteer staffing, the limited clinic resources are often focused on direct clinical services rather than scholarly endeavors [30]. Additional barriers to scholarly work include limitations of personnel bandwidth, lean funding, and minimal research infrastructure. Consequently, many free clinics, including the one described in this study, are unable to engage in research without developing academic partnerships or collaboration with existing research networks. Despite the recognized need for frequent reassessments of SDOH needs and correlations within clinics and local communities to tailor specific, population-centered resources and interventions [15–17], the incentive for in-depth scholarly investigation of uninsured adults at free clinics remains low.

By studying correlations in SDOH and psychosocial resource usefulness at a clinic that exclusively serves uninsured patients, we sought to address these knowledge gaps in this understudied population. This study was conducted at a student-run free clinic in Milwaukee to demonstrate the feasibility of using SDOH screening and longitudinal follow-up data to inform continuous improvements in psychosocial resource offerings and patient care.

## Methods

### Ethical compliance

The Medical College of Wisconsin Institutional Review Board reviewed and granted exempt status for this project (#PRO00028616). As part of clinic workflow, all clinic patients aged 18 years and older were offered to enroll in this study and were provided with informed consent. They did not receive remuneration for their participation. Only those patients who wished to enroll completed the survey (Supplementary Table 1, *N* = 238). Here, we report the findings of survey data which was deidentified prior to analysis. All study procedures were conducted in accordance with the US Federal Regulations for the Protection of Human Subjects.

### Study population

This cross-sectional needs assessment took place at a student-run free clinic in Milwaukee, WI, which primarily serves uninsured adult patients. Clinic patients under 18 years old were excluded from this study. We include information on all participants that completed the SDOH survey (*N* = 238) from October 2021 to October 2022. Patient-reported demographic data was retrieved from clinic intake documentation (Table 1).

**Table 1.** Demographic characteristics among patients screened, including patient-reported race, ethnicity, age, and sex

Characteristic	Total (N = 238)	Percent
**Race**		
Black or African American	87	36.6%
White	53	22.3%
Asian	25	10.5%
Other	19	8.0%
Unknown	6	2.5%
American Indian/Alaska Native	2	0.8%
Native Hawaiian or other Pacific Islander	1	0.4%
Decline to specify	45	18.9%
**Ethnicity**		
Hispanic or Latino	76	31.9%
Not Hispanic or Latino	77	35.7%
Decline to specify	85	32.4%
**Age & Gender**		
**Age Group**	**Male**	**Female**
19–29	8	27
30–39	20	14
40–49	18	30
50–59	21	45
60–69	22	20
70–79	4	6
80+	0	3

### Survey administration

**Figure 1.**
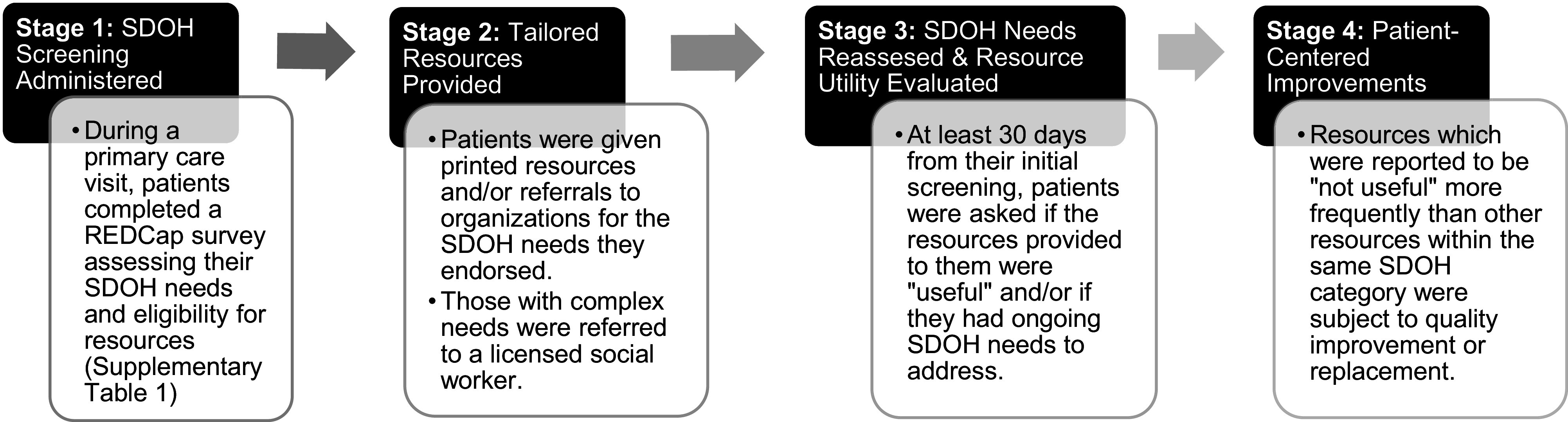
Social determinants of health (SDOH) and health access needs screening process. The four stages of our SDOH screening and resource referral process, which is performed by trained student volunteers for each adult clinic patient.

The research team, clinic social worker, and clinic medical director developed the SDOH survey based on previously conducted research [31,32], observed patient needs, and clinic processes (Supplementary Table 1). The survey was verbally administered by trained resource navigator volunteers during clinic visits (Fig. 1). The resource navigator volunteers were comprised of 2 cohorts of eight unpaid medical students throughout the study period.

Patient responses were recorded in REDCap, an online survey platform that assigns each patient a unique REDCap identifier for longitudinal data collection. The time required for survey administration and assessment of SDOH needs varied based upon patients’ needs. While a full survey with no positive SDOH or access needs took approximately five minutes to complete, patients who endorsed needs were asked further open-ended questions based upon motivational interviewing practices, lengthening time of screening up to 15 minutes total. By administering surveys during preexisting waiting periods within medical visits, such as while a patient was waiting to be seen by a supervising physician or to receive their medications, extra time was not added to patient appointments. Resource navigators also utilized these waiting periods as an opportunity to provide each patient with tailored resources and any associated education regarding access or use of the resources. For patients with complex needs, either beyond the scope of volunteer knowledge or noted to be time intensive (> 1 hr), they were referred to a licensed social worker volunteer within the clinic for additional assistance. For patients with limited English proficiency, volunteers utilized certified telephone medical interpreters to obtain consent and administer the survey.

The survey evaluated patients’ SDOH and access needs, including housing security, utilities, legal assistance, education/work, substance use (smoking, alcohol, and drug use), mental health care access, health insurance, dental care access, food insecurity, and other (Supplementary Table 1). The “other” category was used to solicit any additional patient needs to be addressed such as LGBTQIA + support, women’s health, sexual health, and intimate partner violence. To account for possible interviewer bias, volunteers received synchronous training on resource eligibility criteria and motivational interviewing which was used to guide interview conversations.

The resources provided to patients were curated from an internal resource database, which was audited on a yearly basis to ensure resource offerings and contact information remained updated. Some examples of the 56 local resources listed within the resource database included food pantries, crisis hotlines, legal aid societies, farmer’s market programs, tooth extraction clinics, and employment agencies (Table 2).

**Table 2.** Frequencies of social determinants of health (SDOH) resources distributed and associated patient-reported usefulness at follow-up

A) SDOH Need & Patients Screened Positive (n)	B) Service Type Offered	C) Resource	D) Instances of Resource Distribution (n) *	E) Instances Surveyed About Usefulness (n)	F) Patient(s) Rated Useful †
**Dental Health Access** **(*n* = 154)**	Dental Exam and Cleaning	Dental Cleaning Resource 2	90	9	55.6%
		Dental Cleaning Resource 1	103	31	19.4%
		Dental Cleaning Resource 3	23	8	12.5%
	Dental Procedures	Dental Procedures 1	27	9	0%
		Dental Procedures 2	15	4	0%
	Pediatric Exams & Cleaning	Pediatric Dental Cleaning Resource	37	3	0%
**Insurance** **(*n* = 122)**	Insurance Enrollment Assistance	Insurance Enrollment Agency	61	24	54.2%
	Insurance Coverage	MarketPlace	37	13	61.5%
		Medicare	8	6	50.0%
		Medicaid (BadgerCare)	37	11	45.5%
		Independent Insurance Agent	7	0	N/A
**Work/Education** **(*n* = 57)**	GED and ESL Preparation	Adult Education 1	9	4	50.0%
		Adult Education 2	32	9	22.2%
		Adult Education 3	18	5	20.0%
	Employment Assistance	Employment 1	15	3	66.7%
		Employment 2	17	2	50.0%
	Financial Advising	Financial Advising	2	1	100%
	Job Counseling and Training	Job Training 1	8	0	N/A
	Food Safety Vocational Training	Job Training 2	4	0	N/A
**Utilities** **(*n* = 50)**	Utility Bill Assistance	Utility Assistance 1	33	6	83.3%
	Energy and Water Efficient Fixtures	Utility Assistance 2	20	4	75.0%
	Phone and Internet Assistance	Utility Assistance 3	15	5	60.0%
**Mental Health** **Access** **(*n* = 46)**	Counseling	Mental Health 1	39	12	50.0%
		Mental Health 2	11	3	0%
	Psychotherapy	Mental Health 3	13	3	0%
		Mental Health 4	8	2	0%
	Evaluation & Medication	Mental Health 5	8	3	0%
	Support Groups	Mental Health 6	5	0	N/A
		Mental Health 7	4	0	N/A
	Hotlines	Mental Health Hotline 1	5	0	N/A
		Mental Health Hotline 2	3	0	N/A
		Mental Health Hotline 3	1	0	N/A
		Mental Health Hotline 4	1	0	N/A
**Food** **(*n* = 44)**	Food Provisions	Food Pantries	79	29	65.5%
		Farmers Markets	4	2	0%
		Elderly Food Delivery	2	1	0%
	Financial Assistance for Food	Supplemental Nutrition Assistance Program (SNAP)	26	13	61.5%
		Women, Infants, and Children Program (WIC)	1	0	N/A
	Health Education	Diabetes Advice	6	2	50.0%
		Hypertension Advice	3	3	33.3%
		GERD Advice	4	2	0%
		Nutrition Advice	5	1	0%
		Hyperlipidemia Advice	2	1	0%
**Medication Affordability** **(*n* = 34)**	Discounted Medications	Medications 1	8	1	100%
		Medications 2	16	5	80.0%
		Medications 3	19	9	55.6%
**Legal** **Counseling** **(*n* = 22)**	Immigration Legal Advice	Legal Advice 1	9	2	50.0%
	General Legal Advice	Legal Advice 2	14	5	0%
	IRS & Bankruptcy Legal Advice	Legal Advice 3	2	1	0%
	Civil Legal Advice	Legal Advice 4	4	0	N/A
**Housing** **(*n* = 19)**	Rent Assistance	Rental Assistance	9	1	100%
	Emergency Housing Assistance	Homeless Shelters	5	1	100%
		Women’s Shelters for Domestic Abuse	3	1	0%
	Eviction Legal Support	Eviction Legal Support	1	0	N/A
**Substance** **Abuse** **Treatment** **Access** **(*n* = 18)**	Tobacco Cessation Counseling	Substance Abuse Resource 1	18	7	14.3%
	Medication- Assisted Treatment for Opioid Use Disorder	Substance Abuse Resource 2	1	0	N/A

* Some patients who screen positive for a given SDOH need may receive more than one specific resource addressing that need. Therefore, the summated n for instances of resource distribution (Column D) for a given SDOH need may be larger than the total n who indicated a need (Column A).

† N/A indicates that resource usefulness could not be calculated due to no response data on patient-reported utility after the first year of data collection.

If a patient requested additional assistance navigating a particular resource, they would be scheduled to receive a phone call from a resource navigator within 1–2 weeks to answer further questions and follow-up on barriers to accessing social resources.

All patients presenting for a medical visit were assessed to see if they had completed a recent survey. Patients who had never completed a survey were offered to enroll in the study and complete the survey as noted above. Patients who had completed a survey within the last 30 days were not resurveyed. Patients who last completed a survey 30 or more days ago were asked to repeat the same survey to reassess their SDOH and access needs. Patients who did not return to the clinic for medical visits were not contacted to complete a follow-up survey.

For patients that were resurveyed, resources were provided or reprinted during that clinic visit if new needs were found to be positive or previously identified needs continued to be positive. In addition, resource navigators prompted patients to evaluate previously provided resources’ usefulness on a binary scale of yes or no. Of the 238 patients included in this study, 102 were surveyed at least twice within the study period. The average time between surveys was 80 days, with a standard deviation of 50 days.

### Data analysis

Data analysis was performed using R 4.1.3 (R Foundation for Statistical Computing, Vienna, Austria) with the packages polycor [33], ggplot2 [34], and plotly [35]. To clean survey data, positive and negative responses to survey questions were changed from descriptive values (yes and no) to discreet values (1 and 0). Question 10 (Supplementary Table 1) was coded as 1 for any patient whose food insecurity score was ≥ 2 [32]. For patients who declined to answer or were not asked a given SDOH question, they were coded as a negative response, or value of 0.

Next, positive SDOH needs for any given patient were changed to dichotomous variables based on the principle that if the summation of a given SDOH need was ≥ 1, the patient was assigned a value of 1 for that SDOH need. As a result, patients who screened positive for a given SDOH need during multiple screening instances were not accounted for more than once during analysis.

Tetrachoric correlations (r) were used to assess the strength of pairwise correlation between SDOH needs [36]. Tetrachoric correlations are particularly relevant in scenarios where the observed variables are categorical (binary) but presumed to arise from underlying continuous latent variables. For instance, in our study, the SDOH needs are represented as binary data (present or not present), but they can be conceptualized as manifestations of underlying, more nuanced health-related factors. *P*-values associated with each correlation were adjusted using a Bonferroni correction procedure to reduce potential for a type 1 error (Supplementary Table 2).

## Results

### Demographics

Patients surveyed mostly self-identified as Black or African American (36.6%, *n* = 87) and female (60.9%, *n* = 145), with an average patient age of 48 years (Table 1).

### SDOH needs assessment and correlations

**Figure 2.**
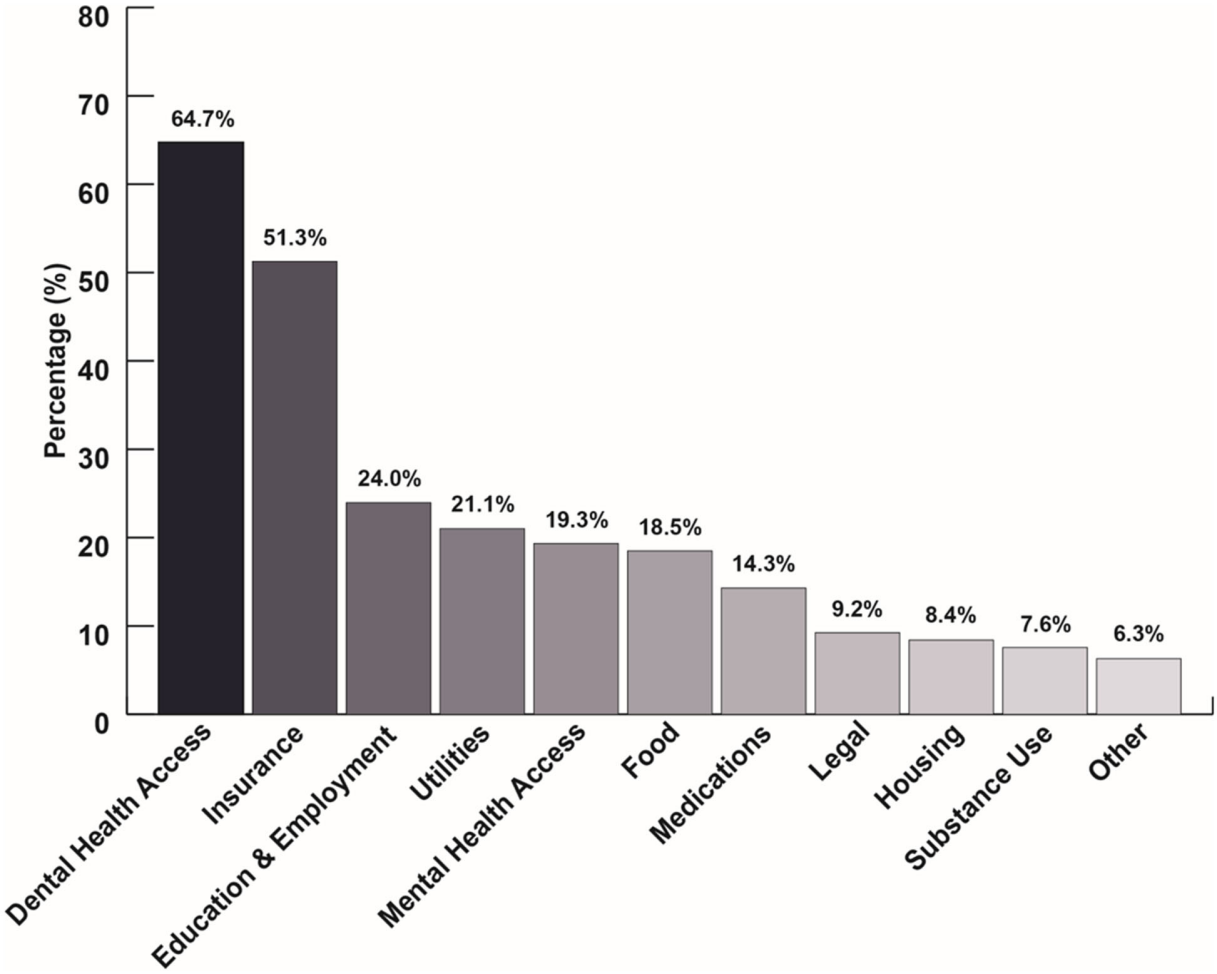
Social determinants of health (SDOH) and health access needs. The percentage distribution of SDOH and health access needs reported by uninsured patients served by a student-run free clinic.

The SDOH and access needs distribution among patients were as follows: dental health access (*n* = 154, 64.7%), health insurance (*n* = 122, 51.3%), education and employment opportunities (*n* = 57, 24.0%), utility assistance (*n* = 50, 21.1%), mental health access (*n* = 46, 19.3%), food insecurity (*n* = 44, 18.5%), medication affordability (*n* = 34, 14.3%), legal assistance (*n* = 22, 9.2%), housing security (*n* = 19, 8.4%), substance use assistance (*n* = 18, 7.6%), and other (*n* = 15, 6.3%) (Fig. 2).

**Figure 3.**
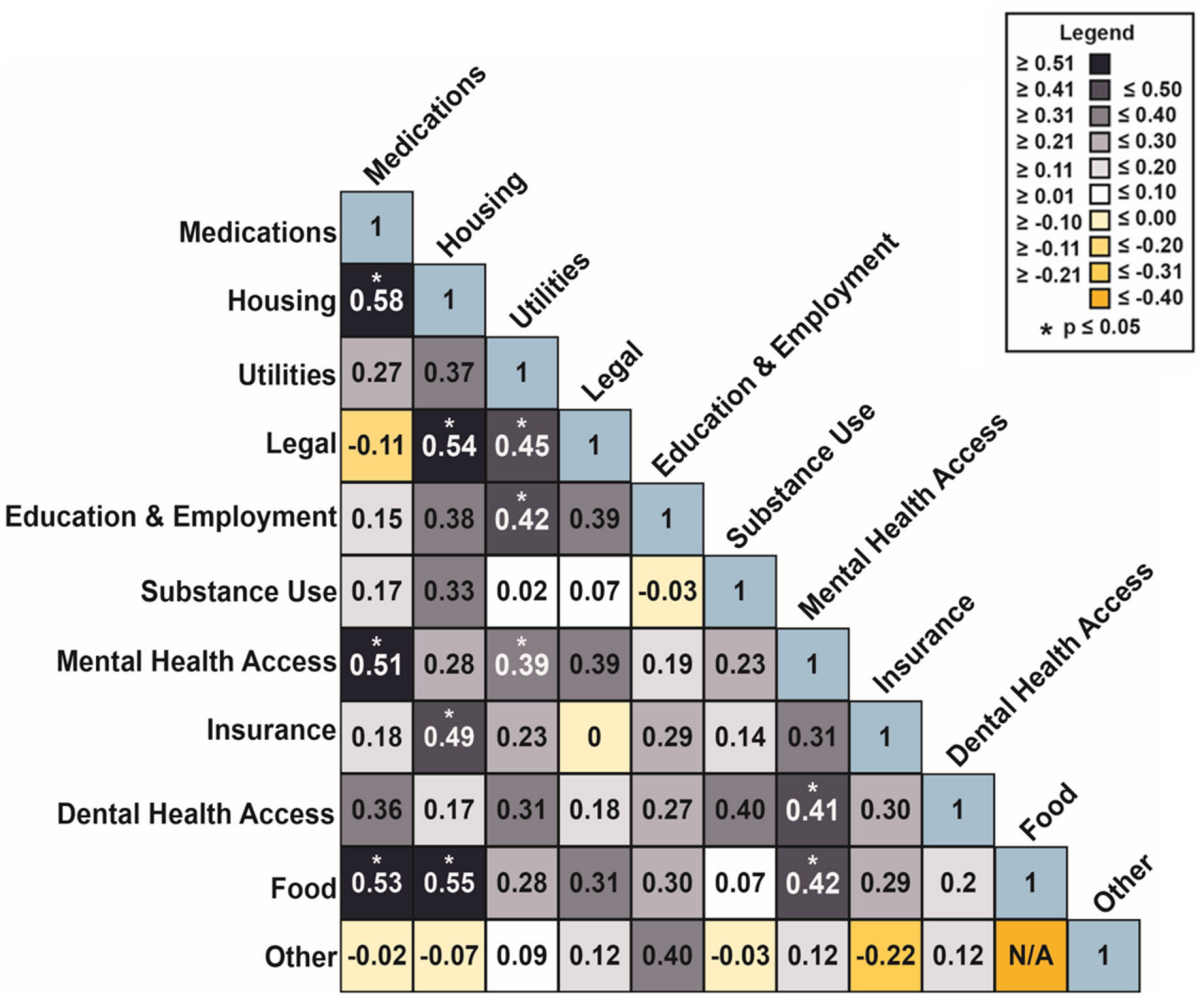
Tetrachoric correlations. A lower triangular matrix of pairwise tetrachoric correlations between social determinants of health access needs. Fill color indicates strength of correlation and significance (*p* ≤ 0.05) is denoted by an asterisk.

We discovered 11 significant correlations (*P* ≤ 0.05) between the SDOH and access needs we assessed. Mental health access needs were correlated with dental health access (*r* = 0.41; 95% CI = 0.19, 0.63), medications (*r* = 0.51; 95% CI = 0.30, 0.72), utilities (*r* = 0.39; 95% CI = 0.17, 0.61), and food insecurity (*r* = 0.42; 95% CI = 0.19, 0.64) (Fig. 3). Furthermore, food insecurity and housing (*r* = 0.55; 95% CI = 0.32, 0.78), housing and medications (*r* = 0.58; 95% CI = 0.35, 0.81), and medications and food insecurity (*r* = 0.53; 95% CI = 0.32, 0.74) were all correlated with each other (Fig. 3). The remaining notable correlations were housing and insurance (*r* = 0.49; 95% CI = 0.24, 0.74), housing and legal (*r* = 0.54; 95% CI = 0.28, 0.81), utilities and education/employment (*r* = 0.42; 95% CI = 0.21, 0.62), and legal and utilities (r = 0.45; 95% CI = 0.20, 0.70) (Fig. 3).

### SDOH resource usefulness

Volunteers curated 56 local resources (community-based organizations, government services, local programs) within the clinic’s internal psychosocial resource database (Table 2). The types of services needed most by patients can be inferred by dividing the instances of resource distribution for a given service type (numerator) by the total instances of resource distribution for a given SDOH (denominator) using Column D of Table 2. For example, within the dental health access category, exams and cleanings were most frequently sought out (73%). Coverage options within insurance-related needs (59%) and GED and ESL preparation within work/education (56%) were also frequently requested resource types (Table 2).

When the instances surveyed about usefulness (Column E) surpassed *n* ≥ 4, patients’ usefulness ratings of resources offering similar services displayed some notable discrepancies (Table 2). For example, “Dental Cleaning Resource 2” was deemed “useful” by 55.6% of rescreened patients, while “Dental Cleaning Resource 1” was only rated “useful” by 19.4%. Within GED and ESL preparation resources, the usefulness of “Adult Education Resource 1” (50.0%) and “Adult Education Resource 2” (22.2%) differed noticeably (Table 2).

## Discussion

This study confirms that an SDOH screening process can provide valuable insight into the needs of patients at a specific clinic. Assessing SDOH and access needs, how they correlate, and patient perspectives on resource usefulness informed clinical practices and resource curation at this Milwaukee free clinic.

### Contextualizing insurance needs

Current literature explores the financial and health benefits of bolstering social services for low-income patient populations but fails to disaggregate the specific benefits for those without insurance [37]. A survey conducted in 2018 found that a third of those who remained uninsured did so because they believed they could not afford insurance and a third of those who lost previous coverage cited affordability concerns as the reason they were no longer insured [38]. This same study also suggested an overall lack of knowledge about coverage options and eligibility for expanded Medicaid or Marketplace tax credits [38]. Providing social services which address needs related to employment, housing, utilities, medications, and food may allow patients to reallocate their available income towards affording insurance and avoiding unaffordable emergency medical bills. This must be done in tandem which increased education about insurance eligibility to target the aforementioned lack of knowledge.

Additionally, being uninsured may impact how patients access other social services. Many social services include insurance status as part of their eligibility criteria, presenting uninsured patients with unique barriers. Acknowledging these barriers, at the free clinic where this study was conducted, social service resources were selected and provided in a way that served our uninsured patient population. Additionally, since accessing health insurance was a screened-for need, this clinic made sure to understand the context of why each patient was uninsured and consider how those circumstances might intersect with their inability to access other social services. It was arguably predictable that 51.3% patients surveyed at a clinic for uninsured individuals sought resources for health insurance. However, volunteers were frequently asked to clarify insurance options, eligibility, or enrollment processes, thus proving the utility of continuing to screen for this SDOH and monitor patient feedback about resources. Of those who did not request insurance resources, some anecdotally cited that primary care visits at our clinic addressed their needs or that they already understood existing health insurance options were not accessible due to eligibility criteria or affordability.

Many SDOH intervention platforms, such as Impact Connect and FindHelp, measure the success of resource referrals by surveying community partners’ receipt of new patients [39,40]. Our process for resource evaluation, rooted in patient-reported data, centers the patient voice rather than the voices of community-based organizations. For example, the only local resource for insurance enrollment assistance that counsels patients on plans and eligibility, referred to as “Insurance Enrollment Agency,” was deemed useful by our patients 54.2% of the time (Table 2). In response, the research team developed a new clinic volunteer role of “Health Insurance Enrollment Assistant” to provide patients with individualized education and application assistance during in-person clinic visits.

### Contextualizing dental health access

In our patient population, the most prominent surveyed need was dental health access. To investigate the low usefulness rating of Dental Cleaning Resource 1 (19.4%), the research team reached out to the clinic staff at “Dental Cleaning Resource 1” and discovered that staffing shortages limited their capacity for new patients. No patients or staff from this community-based dental clinic had relayed this information to our clinic thus far, so without the survey’s data we would have likely continued referring patients to this local dental clinic with minimal availability. Patient feedback incentivized the identification of alternative resources. We began preferentially providing patients with “Dental Cleaning Resource 2” instead of “Dental Cleaning Resource 1,” which garnered a comparatively higher patient-reported usefulness (55.6%, Table 2). This modification exemplifies how patient-centered data improved resource curation by leading to the prioritization of a resource with a greater capacity for new patients.

The highest usefulness rating that any dental resource reached was 55.6% (“Dental Cleaning Resource 2”). This relatively low rating, despite its improvement over “Dental Cleaning Resource 1,” may suggest that a gap exists in high-quality dental services for uninsured patients in the Milwaukee area. Other barriers which could have decreased patients’ usefulness ratings include transportation or scheduling constraints.

### Contextualizing needs correlated with mental health access

In this patient population, mental health access needs correlated with the greatest number of other SDOH needs (dental, medications, utilities, and food). Our finding of the correlation between mental and dental health access corroborates existing literature findings [2–4]. Previous research suggests the following rationales for the strong relationship between these two social determinants: anhedonia related to depression, underutilization of dental clinics due to barriers, and poor interprofessional integration between mental health and dental services [2–4]. By training volunteers to facilitate dental care referrals during primary health care visits, our clinic sought to ameliorate root causes of the two correlated needs. Additionally, some medications prescribed for psychiatric illnesses are associated with oral side effects, such as xerostomia, which may increase the prevalence of dental disease in patients with mental illness [41]. While clinic volunteers currently give all patients free dental hygiene supplies at every visit, we are tailoring additional education for patients regarding the oral side effects of psychiatric medications moving forward.

Medications, utilities, and food also significantly correlated with poor mental health care access. Previous research has linked food insecurity to worse mental health outcomes [5,42], and this study discovered a similar correlation, specifically in the uninsured population. To comprehensively address concurrent SDOH needs, clinic volunteers provide patients with resources to reduce utility costs which may increase financial reserve for food. The clinic also ensures that patients receive free, in-house prescriptions.

### Contextualizing correlations between housing, medication, and food

Housing, medications, and food all significantly correlated with each other, which may be explained by a root cause of financial stress (Fig. 3). The most frequently distributed housing resources offered rental assistance (50%) highlighting that economic instability could be underlying housing needs (Table 2). Additionally, the survey prompts administrators to directly ask about medication accessibility and food security through a lens of affordability (Supplementary Table 1). Thus, financial instability likely explains the correlations between these three SDOH needs. Housing, food, and medication costs can be competing monetary priorities. Based on this understanding, large public benefit systems should prioritize patient resources which directly ameliorate financial instability [43].

The remaining significant correlations in this study were housing and insurance, housing and legal, utilities and education/employment, and legal and utilities (Fig. 3). The association between housing instability and other SDOH needs is supported by previous research postulating that having safe, stable housing is essential to maintaining an individual’s overall health and well-being [44].

### Feasibility

Perhaps one of the strongest contributions of this study is its demonstration of the feasibility of assessing and addressing SDOH in a student-run free clinic setting. Due to budget constraints, our free clinic opted for an EHR software that did not include pre-configured provider orders and web-based referrals. To overcome this financial barrier, we implemented an EHR-agnostic SDOH screening by conducting the survey through REDCap, a free, secure and HIPAA compliant software accessible to any non-profit organization [45,46]. For free clinics seeking to adopt this survey methodology, we are prepared to share the foundational code required for implementing a comparable SDOH screening process via REDCap.

This project’s success relied on the generous time and energy of willing student volunteers. Clinic leaders developed a curriculum to train selected volunteers as resource navigators that could administer SDOH screenings and provide tailored resources. These trainings could be adapted for volunteers at other free clinics. Given the low cost associated with this method, both in terms of resources and labor, it can accessibly be implemented in other clinical settings.

The multidisciplinary workflow of the clinic allowed patients to be surveyed while they were awaiting assessment by their provider team or while waiting for their in-house prescriptions to be prepared. Patients were always surveyed before their medical visit ended so that they did not have to extend their appointment to complete the survey. Most patients consented to be surveyed and often expressed gratitude for the resources provided to address various SDOH needs.

One of the few patient critiques received about the survey was made by patients who visited the clinic more frequently. They shared that being surveyed after 30 days was too short of a time interval to have utilized the SDOH resources provided. Therefore, it would be acceptable to consider extending the amount of time between surveys to give patients more time to access the resources provided and decrease survey burden/fatigue.

### Impact on clinical practice and resource curation

Overall, this SDOH screening process and respective findings enabled us to implement changes to clinical service and improve psychosocial resource curation based on patient-centered findings. After recognizing the correlation between mental and dental health needs, our team began providing tailored counseling to patients seen for psychiatric conditions. In response to seeing food insecurity’s correlations with numerous other SDOH, clinic volunteers began to provide stock bags of nonperishable food items from local pantries for patients who demonstrated this need. As already detailed in Sections 4.1 and 4.2, patient-reported usefulness led to the creation of a new “Health Insurance Enrollment Assistant” volunteer role and the addition of “Dental Cleaning Resource 2” in our internal resource database.

As we collect additional responses and continue assessing trends, we are considering several interventions to further address clinic patients’ SDOH and health access needs. For example, we are investigating the discrepancy in usefulness of adult education resources and gauging whether the new, in-house “Health Insurance Enrollment Assistant” volunteers provided greater health insurance support than previously distributed external resources. Building upon this initial analysis, we look forward to using resource usefulness ratings to inform distribution practices and guide annual resource audits.

### Study limitations

The data used in this study was collected over a relatively short period of time, limiting the extent to which claims can be made regarding patient SDOH needs over the course of multiple clinic visits. Using tetrachoric correlations, relationships between several variables were established, but it was not possible to analyze causality. Only a small number of patients were longitudinally surveyed about usefulness of previously provided resources, and additional study is needed for more comprehensive interpretation. Furthermore, the COVID-19 pandemic may have impacted patients’ ability to access resources.

Although the direct costs associated with this SDOH screening implementation in other clinic settings is low, both in terms of resources and labor, this model was dependent on the generous time of medical students, physicians, and volunteers. While volunteers help save resource-limited clinics money, the cost of training and supporting volunteers is not to be dismissed.

## Conclusion

This study analyzed SDOH needs, health access needs, and their correlations within an understudied, uninsured population and assessed the usefulness of resources based on patient-reported longitudinal data. This data subsequently informed resource distribution and resource bank curation. The success of this SDOH screening process in a student-run clinic demonstrates its applicability to other low-resource clinics. This cross-sectional study provides a proof of concept for screening, tracking, and addressing SDOH in an uninsured patient population at a student-run free clinic. This data collection method can serve a shared goal among free and charitable health clinics: improving the health of their community. It can also provide strong, research supported practices that can advocate for policy changes, which truly address health inequity.

## Supporting information

Miller et al. supplementary material Miller et al. supplementary material

## Data Availability

The data that support the findings of this study are available on request from the corresponding authors, J.M. or A.D. The data are not publicly available to support patient privacy. For free clinics seeking to adopt comparable SDOH screening, please reach out to the corresponding authors to obtain the foundational REDCap code.

## Supplementary material

The supplementary material for this article can be found at https://doi.org/10.1017/cts.2024.503.
