# Supplementary material for: Social determinants of health correlations and resource usefulness at a Milwaukee free clinic for uninsured individuals: A cross-sectional study
Source: J Clin Transl Sci. 2024 Apr 25;8(1):e71. doi: 10.1017/cts.2024.503 (PMC11058579; doi:10.1017/cts.2024.503)
Supplement: Miller et al. supplementary material [file S205986612400503Xsup001.docx]

**Supplementary Table 1: Social Determinants of Health (SDOH) REDCap Survey Administered**

| **SDOH Survey Script (Administered Verbally Using REDCap)** | |
| --- | --- |
| **The Saturday Clinic for the Uninsured has information on many health-related services. We know that factors at home may influence your ability to be healthy. As part of your visit, we'd like to ask you a few questions related to social factors which may impact your health and provide resources to help you maintain your health. This survey is optional. You may complete the whole survey or parts of the survey. If you choose to complete this survey, your responses are confidential and protected.** | |
| **Survey Question** | **Answer Options** |
| 1) In the last 12 months, did you ever skip medications to save money? | Yes |
|  | No |
|  | Prefer not to answer |
|  | Question Not Asked |
| 2) In the next two months, are you worried you may not have stable housing? | Yes |
|  | No |
|  | Prefer not to answer |
|  | Question Not Asked |
| 3) Do you need assistance with paying for or lowering utility bills? This could include energy, phone, water, or Wi-Fi. | Yes |
|  | No |
|  | Prefer not to answer |
|  | Question Not Asked |
| 4) Do you have questions about legal issues related to immigration, child support, or eviction? | Yes |
|  | No |
|  | Prefer not to answer |
|  | Question Not Asked |
| 5) Would you be interested in learning about educational or work opportunities? | Yes |
|  | No |
|  | Prefer not to answer |
|  | Question Not Asked |
| 6) Is there a substance use problem or addiction that you would like resources about today? For example, assistance with quitting smoking, alcohol, or drug use. | Yes |
|  | No |
|  | Prefer not to answer |
|  | Question Not Asked |
| 7) Are you interested in counseling or mental health services? | Yes |
|  | No |
|  | Prefer not to answer |
|  | Question Not Asked |
| 8) Would you like information about health insurance options you qualify for? | Yes |
|  | No |
|  | Prefer not to answer |
|  | Question Not Asked |
| 9) Would you like to be connected with a free or low-cost dentist for teeth cleaning or other dental problems? | Yes |
|  | No |
|  | Prefer not to answer |
|  | Question Not Asked |
| 10a) The food that we bought just didn’t last, and we didn’t have money to get more. | Often |
|  | Sometimes |
|  | Never |
| 10b) We couldn't afford to eat balanced meals. | Often |
|  | Sometimes |
|  | Never |
| 10c) In the last 30 days, did you or other adults in your household ever cut the size of your meals or skip meals because there wasn't enough money for food? | Yes |
|  | No |
|  | Prefer not to answer |
|  | Question Not Asked |
| 10c) (IF YES PRIOR QUESTION) In the last 30 days, how many days did this happen? | Patient response |
| 10d) In the last 30 days, did you ever eat less than you felt you should because there wasn't enough money for food? | Yes |
|  | No |
|  | Prefer not to answer |
|  | Question Not Asked |
| 10e) In the last 30 days, were you ever hungry but didn't eat because there wasn't enough money for food? | Yes |
|  | No |
|  | Prefer not to answer |
|  | Question Not Asked |
| 11) Do you have any other concerns you would like to discuss or resources you may need? (Ex. Women's Health, LGBTQ community, safe sex practices, sexual health, intimate partner violence, domestic violence) | Yes |
|  | No |
|  | Prefer not to answer |
|  | Question Not Asked |

**Supplementary Table 2: Pairwise Relationship Between Social Determinants of Health (SDOH) and Associated Tetrachoric P Values Generated by Bonferroni Correction**

| **SDOH Category** | **P Adjusted** | **R Value** | **95% CI Low er** | **95% CI Upper** |
| --- | --- | --- | --- | --- |
| Housing & Medications | 0.000038 | 0.58 | 0.35 | 0.81 |
| Medications & Food | 0.000043 | 0.53 | 0.32 | 0.74 |
| Mental Health & Medications | 0.00016 | 0.51 | 0.30 | 0.72 |
| Food & Housing | 0.00020 | 0.55 | 0.32 | 0.78 |
| Housing & Legal | 0.0032 | 0.54 | 0.28 | 0.81 |
| Utilities & Education/Employment | 0.0051 | 0.42 | 0.21 | 0.62 |
| Housing & Insurance | 0.0084 | 0.49 | 0.24 | 0.74 |
| Mental Health & Dental | 0.012 | 0.41 | 0.19 | 0.63 |
| Mental Health & Food | 0.013 | 0.42 | 0.19 | 0.64 |
| Legal & Utilities | 0.020 | 0.45 | 0.20 | 0.70 |
| Mental Health & Utilities | 0.033 | 0.39 | 0.17 | 0.61 |
| Legal & Mental Health | 0.063 | 0.43 | 0.17 | 0.68 |
| Insurance & Dental | 0.084 | 0.31 | 0.12 | 0.50 |
| Medications & Dental | 0.096 | 0.38 | 0.14 | 0.63 |
| Legal & Education/Employment | 0.13 | 0.40 | 0.14 | 0.65 |
| Mental Health & Insurance | 0.17 | 0.32 | 0.11 | 0.54 |
| Housing & Education/Employment | 0.23 | 0.39 | 0.12 | 0.65 |
| Education/Employment & Insurance | 0.26 | 0.30 | 0.091 | 0.53 |
| Utilities & Dental | 0.29 | 0.31 | 0.094 | 0.54 |
| Education/Employment & Other | 0.31 | 0.40 | 0.12 | 0.68 |
| Housing & Utilities | 0.36 | 0.38 | 0.11 | 0.65 |
| Insurance & Food | 0.46 | 0.30 | 0.076 | 0.51 |
| Substance Use & Dental | 0.47 | 0.40 | 0.10 | 0.70 |
| Education/Employment & Food | 0.62 | 0.30 | 0.068 | 0.53 |
| Education/Employment & Dental | 0.86 | 0.27 | 0.051 | 0.49 |
| Utilities & Food | 1 | 0.28 | 0.047 | 0.52 |
| Legal & Food | 1 | 0.31 | 0.034 | 0.60 |
| Medications & Utilities | 1 | 0.28 | 0.028 | 0.53 |
| Utilities & Insurance | 1 | 0.23 | 0.016 | 0.45 |
| Housing & Substance Use | 1 | 0.35 | 0.0088 | 0.69 |
| Housing & Mental Health | 1 | 0.27 | -0.025 | 0.56 |
| Dental & Food | 1 | 0.20 | -0.037 | 0.43 |
| Education/Employment & Mental Health | 1 | 0.19 | -0.055 | 0.43 |
| Substance Use & Mental Health | 1 | 0.24 | -0.071 | 0.54 |
| Insurance & Other | 1 | -0.23 | -0.53 | 0.073 |
| Legal & Dental | 1 | 0.20 | -0.087 | 0.49 |
| Medications & Insurance | 1 | 0.17 | -0.076 | 0.41 |
| Medications & Education/Employment | 1 | 0.16 | -0.098 | 0.42 |
| Housing & Dental | 1 | 0.16 | -0.14 | 0.45 |
| Medications & Substance Use | 1 | 0.17 | -0.17 | 0.51 |
| Substance Use & Insurance | 1 | 0.13 | -0.16 | 0.43 |
| Medications & Legal | 1 | -0.14 | -0.50 | 0.22 |
| Dental & Other | 1 | 0.12 | -0.20 | 0.45 |
| Mental Health & Other | 1 | 0.13 | -0.22 | 0.47 |
| Utilities & Other | 1 | 0.095 | -0.25 | 0.44 |
| Legal & Other | 1 | 0.11 | -0.30 | 0.52 |
| Substance Use & Food | 1 | 0.071 | -0.26 | 0.41 |
| *Food & Other | N/A | N/A | N/A | N/A |
| Legal & Substance Use | 1 | 0.057 | -0.34 | 0.46 |
| Housing & Other | 1 | -0.061 | -0.53 | 0.40 |
| Education/Employment & Substance Use | 1 | -0.030 | -0.36 | 0.30 |
| Substance Use & Other | 1 | -0.033 | -0.51 | 0.44 |
| Utilities & Substance Use | 1 | 0.022 | -0.31 | 0.35 |
| Legal & Insurance | 1 | -0.018 | -0.30 | 0.26 |
| Medications & Other | 1 | -0.022 | -0.41 | 0.37 |
| ***The confidence interval cannot be estimated for the pair of "food & other" statistically due to a zero within the contingency table** | | | | |
